# Supplementary material for: The evolution of antimicrobial peptide resistance in Pseudomonas aeruginosa is severely constrained by random peptide mixtures
Source: PLoS Biol. 2024 Jul 2;22(7):e3002692. doi: 10.1371/journal.pbio.3002692 (PMC11218975; doi:10.1371/journal.pbio.3002692)
Supplement: S1 Table — (DOCX) [file pbio.3002692.s001.docx]

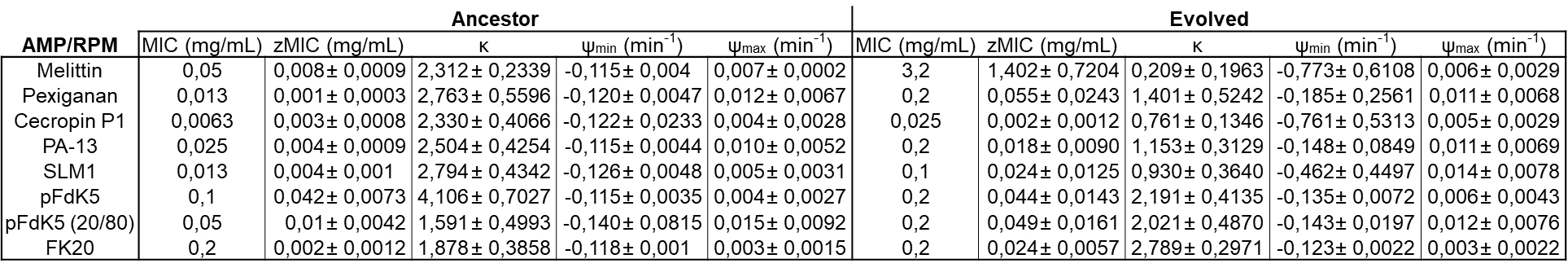


*Table S1 – Pharmacodynamic parameters (zMIC, κ, ψ_min_ and ψ_max_) for the ancestor and most resistant evolved strains, acquired by fitting time-kill curves to the Hill function using Rstan.*
