# Supplementary material for: The evolution of antimicrobial peptide resistance in Pseudomonas aeruginosa is severely constrained by random peptide mixtures
Source: PLoS Biol. 2024 Jul 2;22(7):e3002692. doi: 10.1371/journal.pbio.3002692 (PMC11218975; doi:10.1371/journal.pbio.3002692)
Supplement: S2 Table — One model was fitted to each gene/antimicrobial combination. The explanatory variable is the presence, or absence, of a SNP in the focal gene (regardless of the selection regime the strain originated from), and the response variable is the MIC fold-change to the various antimicrobials of interest: presence/absence of SNPs in gene~MIC fold-change. The Chi square with degrees of freedom (X2df,ddf) is given on top, the p-value on the bottom. The cells of the table containing no results (-) are the combinations of SNP and antimicrobial for which there was only 1 bacterial strain showing no SNP in the given gene, therefore not allowing for a proper comparison. The models for which the presence of SNP in the focal gene significantly influenced the MIC fold-change have their results written in bold. (DOCX) [file pbio.3002692.s002.docx]

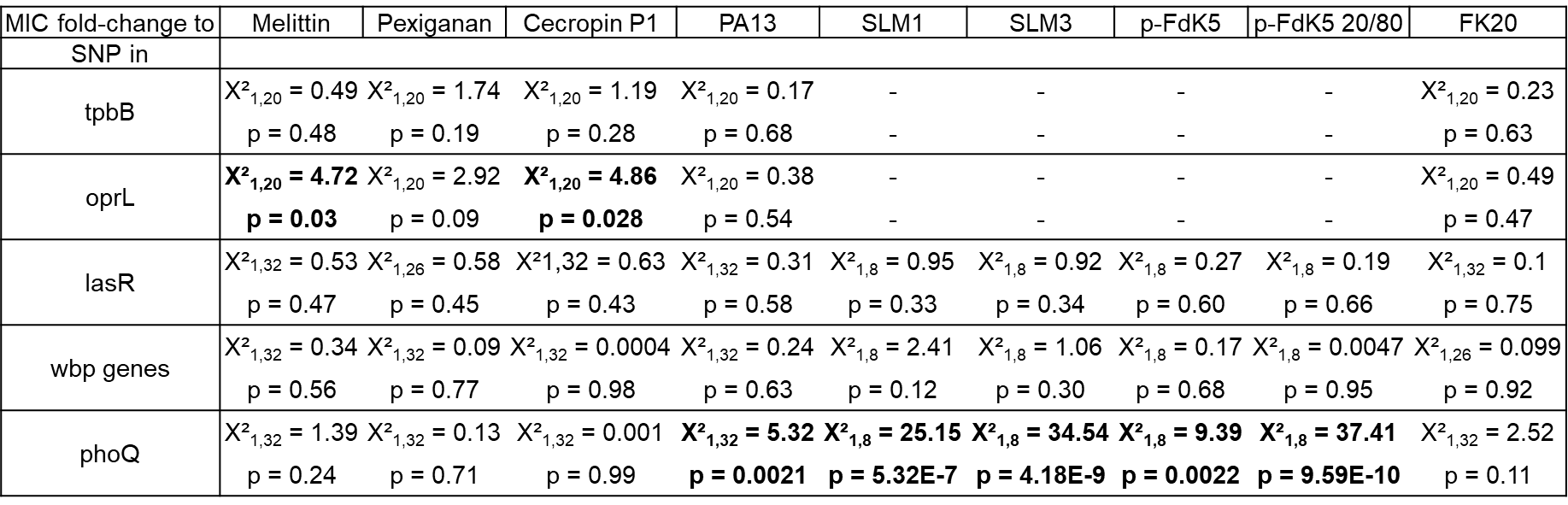


*Table S2 – Summary of the results of the GLMMs (GLMs in the case of the tpbB and oprL genes, for which SNPs were present only in 1 replicate of the experiment, thus not requiring a random factor), performed across strains of several selection regimes, by testing the influence of SNPs in several genes on the magnitude of resistance evolution. One model was fitted to each gene/antimicrobial combination. The explanatory variable is the presence, or absence, of a SNP in the focal gene (regardless of the selection regime the strain originated from), and the response variable is the MIC fold-change to the various antimicrobials of interest: presence/absence of SNPs in gene~MIC fold-change. The Chi square with degrees of freedom (X²df,ddf) is given on top, the p-value on the bottom. The cells of the table containing no results (-) are the combinations of SNP and antimicrobial for which there was only 1 bacterial strain showing no SNP in the given gene, therefore not allowing for a proper comparison. The models for which the presence of SNP in the focal gene significantly influenced the MIC fold-change have their results written in bold.*
