# Supplementary material for: The evolution of antimicrobial peptide resistance in Pseudomonas aeruginosa is severely constrained by random peptide mixtures
Source: PLoS Biol. 2024 Jul 2;22(7):e3002692. doi: 10.1371/journal.pbio.3002692 (PMC11218975; doi:10.1371/journal.pbio.3002692)
Supplement: S1 Fig — OD595 was measured every 15 min through 24 h. (A) Lag time (n = 6; X29,40 = 44,31; p < 0.0001); (B) maximum growth rate (Vmax; n = 6; X29,40 = 11,9; p = 0.22). The boxes span the range between the 25th and 75th percentile, while the horizontal black line inside represents the median value. The vertical bars extend to the minimum and maximum score, excluding outliers. The results represent 2 independent experiments. The data underlying this figure can be found in https://doi.org/10.5281/zenodo.11209304. (DOCX) [file pbio.3002692.s003.docx]

*Figure S1 – Fitness cost of individual peptide evolved strains, determined by growing the bacteria in the absence of AMPs and normalised to that of the ancestor. OD_595_ was measured every 15 min through 24 h. (A) Lag time (n=6; X²_9,40_ = 44,31; p<0.0001); (B) Maximum growth rate (Vmax; n=6; X²_9,40_ = 11,9; p=0.22). The boxes span the range between the 25th and 75th percentile, while the horizontal black line inside represents the median value. The vertical bars extend to the minimum and maximum score, excluding outliers. The results represent two independent experiments. The data underlying this Figure can be found in* <https://doi.org/10.5281/zenodo.11209304>*.*
