# Supplementary material for: The evolution of antimicrobial peptide resistance in Pseudomonas aeruginosa is severely constrained by random peptide mixtures
Source: PLoS Biol. 2024 Jul 2;22(7):e3002692. doi: 10.1371/journal.pbio.3002692 (PMC11218975; doi:10.1371/journal.pbio.3002692)
Supplement: S2 Fig — (DOCX) [file pbio.3002692.s004.docx]

*Figure S2 – Heat map of mutations obtained from whole-genome sequencing, using the ancestor PA14 as reference.*
