# Supplementary material for: The evolution of antimicrobial peptide resistance in Pseudomonas aeruginosa is severely constrained by random peptide mixtures
Source: PLoS Biol. 2024 Jul 2;22(7):e3002692. doi: 10.1371/journal.pbio.3002692 (PMC11218975; doi:10.1371/journal.pbio.3002692)
Supplement: S3 Fig — Resistance determined by MIC assay of each strain toward the corresponding peptide. Results shown as log2 fold-change of the ancestor MICs. Each dot represents the mean of triplicates. The data underlying this figure can be found in https://doi.org/10.5281/zenodo.11209304. (DOCX) [file pbio.3002692.s005.docx]

*Figure S3 – Relation between resistance evolution (MIC fold-change) and all gene mutations assessed by whole-genome sequencing. Resistance determined by MIC assay of each strain toward the corresponding peptide. Results shown as log2 fold-change of the ancestor MICs; Each dot represents the mean of triplicates. The data underlying this Figure can be found in* <https://doi.org/10.5281/zenodo.11209304>*.*
