# Supplementary material for: The evolution of antimicrobial peptide resistance in Pseudomonas aeruginosa is severely constrained by random peptide mixtures
Source: PLoS Biol. 2024 Jul 2;22(7):e3002692. doi: 10.1371/journal.pbio.3002692 (PMC11218975; doi:10.1371/journal.pbio.3002692)
Supplement: S4 Fig — Resistance determined by MIC assay of each strain toward the corresponding peptide. Results shown as log2 fold-change of the ancestor MICs. Each dot represents the mean of triplicates (values beside the dots represent the MIC value). The x-axis displays individual strains per treatment and the respective mutations (or absence of) are portrayed underneath. The results represent 2 independent experiments. The data underlying this figure can be found in https://doi.org/10.5281/zenodo.11209304. (DOCX) [file pbio.3002692.s006.docx]

*Figure S4 – Representation of the resistance evolution of control strains (MIC fold-changes) in relation to the presence/absence of mutations in the 5 most frequently mutated genes. Resistance determined by MIC assay of each strain toward the corresponding peptide. Results shown as log2 fold-change of the ancestor MICs; Each dot represents the mean of triplicates (values beside the dots represent the MIC value). The x-axis displays individual strains per treatment and the respective mutations (or absence of) are portrayed underneath. The results represent two independent experiments. The data underlying this Figure can be found in* <https://doi.org/10.5281/zenodo.11209304>*.*
