# Supplementary material for: The evolution of antimicrobial peptide resistance in Pseudomonas aeruginosa is severely constrained by random peptide mixtures
Source: PLoS Biol. 2024 Jul 2;22(7):e3002692. doi: 10.1371/journal.pbio.3002692 (PMC11218975; doi:10.1371/journal.pbio.3002692)
Supplement: S5 Fig — The x-axis represents the antimicrobial used as a selection pressure during experimental evolution of the bacterial strains, as well as for assessment of their MIC fold-change (see colour code in the guide on the right side of the plot). The y-axis is a non-linear scale of the MIC fold-change multiplied by 1,000, to be integer and satisfy assumptions for a negative-binomial GLMM. Full dots represent coefficients for the strain selected against the assessed antimicrobial, whereas open dots represent the coefficient for the control strain, serially passaged in the absence of antimicrobial and tested against the antimicrobial of interest. A significant difference between treatment levels is observed when 95CI do not overlap on more than half of their length (see “Statistical analysis” section). The data underlying this figure can be found in https://doi.org/10.5281/zenodo.11209304. (DOCX) [file pbio.3002692.s007.docx]

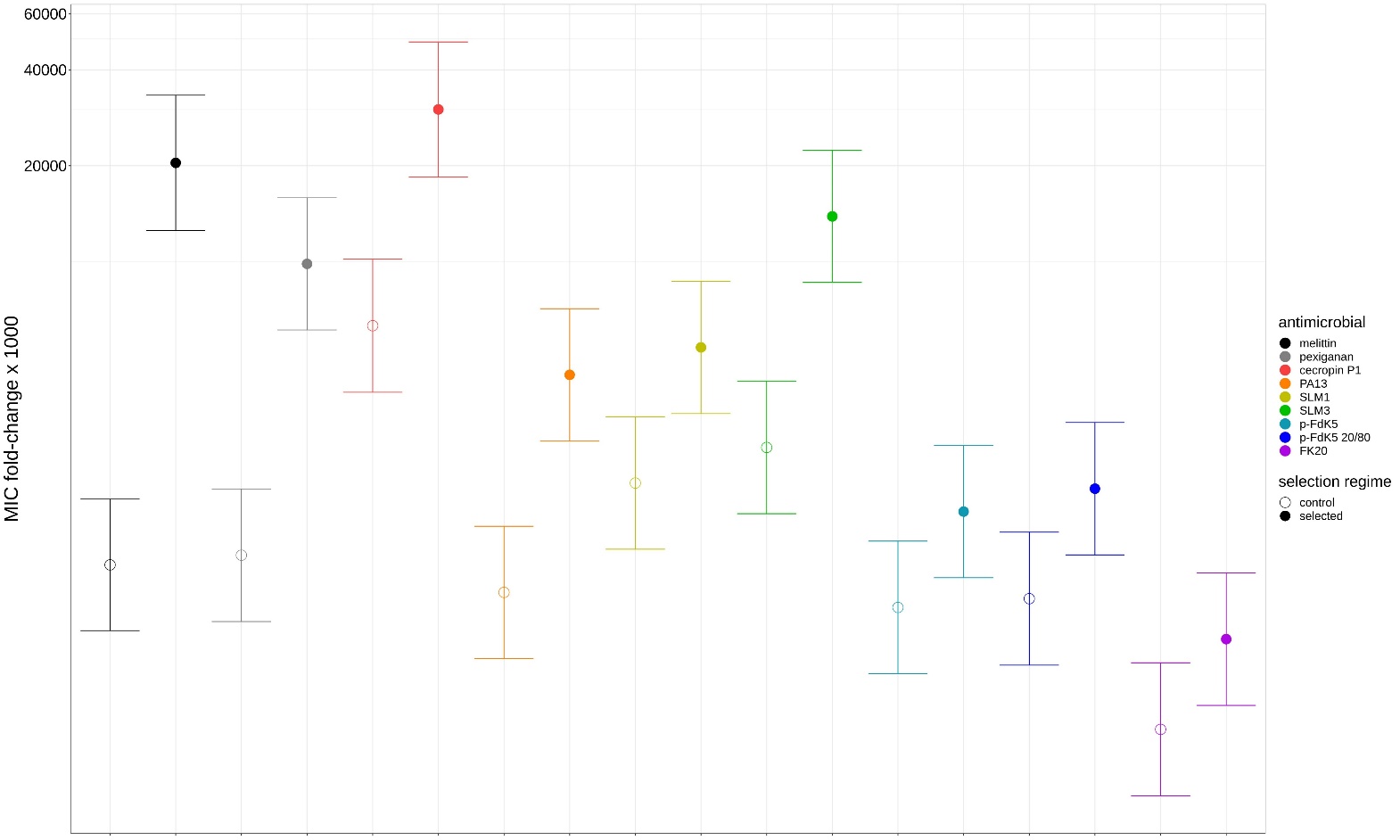


*Figure S5 – Plot displaying the coefficients (dots) with 95CIs (vertical bars) of the negative binomial GLMM analysing MIC fold-change, according to the selection regime as a focal predictor, including an experimental block as a random factor. The x axis represents the antimicrobial used as a selection pressure during experimental evolution of the bacterial strains, as well as for assessment of their MIC fold-change (see colour code in the guide on the right side of the plot). The y axis is a non-linear scale of the MIC fold-change multiplied by 1000, to be integer and satisfy assumptions for a negative-binomial GLMM. Full dots represent coefficients for the strain selected against the assessed antimicrobial, whereas open dots represent the coefficient for the control strain, serially passaged in the absence of antimicrobial and tested against the antimicrobial of interest. A significant difference between treatment levels is observed when 95CI do not overlap on more than half of their length (see ‘Statistical analysis’ section). The data underlying this Figure can be found in* <https://doi.org/10.5281/zenodo.11209304>*.*
