# Supplementary material for: The evolution of antimicrobial peptide resistance in Pseudomonas aeruginosa is severely constrained by random peptide mixtures
Source: PLoS Biol. 2024 Jul 2;22(7):e3002692. doi: 10.1371/journal.pbio.3002692 (PMC11218975; doi:10.1371/journal.pbio.3002692)
Supplement: S7 Fig — The x-axis represents the antimicrobial used as a selection pressure during experimental evolution of the bacterial strains (see colour code in the guide on the right side of the plot). The y-axis represents the Vmax fold-change. A significant difference between treatment levels is observed when 95CI do not overlap on more than half of their length (see “Statistical analysis” section). The data underlying this figure can be found in https://doi.org/10.5281/zenodo.11209304. (DOCX) [file pbio.3002692.s009.docx]

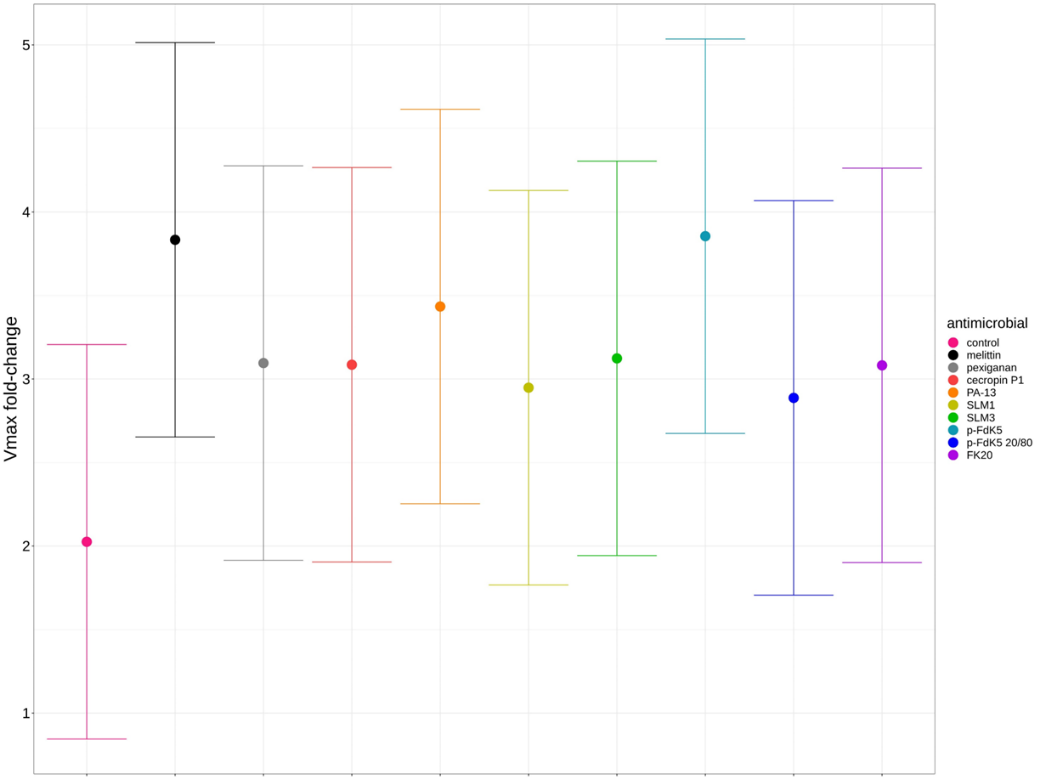


*Figure S7 – Plot displaying the coefficients (dots) with 95CIs (vertical bars) of the LMM analysing the Vmax fold-change (in the absence of antimicrobial), according to the selection regime as a focal predictor, including an experimental block as a random factor. The x axis represents the antimicrobial used as a selection pressure during experimental evolution of the bacterial strains (see colour code in the guide on the right side of the plot). The y axis represents the Vmax fold-change. A significant difference between treatment levels is observed when 95CI do not overlap on more than half of their length (see ‘Statistical analysis’ section). The data underlying this Figure can be found in* <https://doi.org/10.5281/zenodo.11209304>*.*
