# Supplementary material for: The evolution of antimicrobial peptide resistance in Pseudomonas aeruginosa is severely constrained by random peptide mixtures
Source: PLoS Biol. 2024 Jul 2;22(7):e3002692. doi: 10.1371/journal.pbio.3002692 (PMC11218975; doi:10.1371/journal.pbio.3002692)
Supplement: S9 Fig — The antimicrobial to which the bacterial strains were exposed to, is given in the window of the boxplot. On the x-axis: absence (0) or presence (1) of oprL gene SNPs. On the y-axis: MIC fold-change of experimental evolution. The boxes span the range between the 25th and 75th percentile, while the horizontal black line inside represents the median value. The vertical bars extend to the minimum and maximum score, excluding outliers. The individual datapoints represent the MIC fold-change of 1 bacterial strain carrying an SNP. SNPs in the tpbB gene emerged only in 1 replicate of the experiment. The boxplots representing MIC fold changes towards Melittin, Pexiganan, Cecropin P1, PA13, and FK20 contain the data of strains originating from the Melittin, Pexiganan, Cecropin P1, PA13, and control selection regimes. The boxplots representing MIC fold-change towards SLM1, SLM3, p-FdK5, and p-FdK5 20/80 contain the data of strains originating from the SLM1 and control treatments. In the SLM1 selection regime, only 1 strain did not show an SNP in oprL, making the comparison between presence/absence of SNP irrelevant. The corresponding statistical tests are given in the S2 Table. The data underlying this figure can be found in https://doi.org/10.5281/zenodo.11209304. (DOCX) [file pbio.3002692.s011.docx]

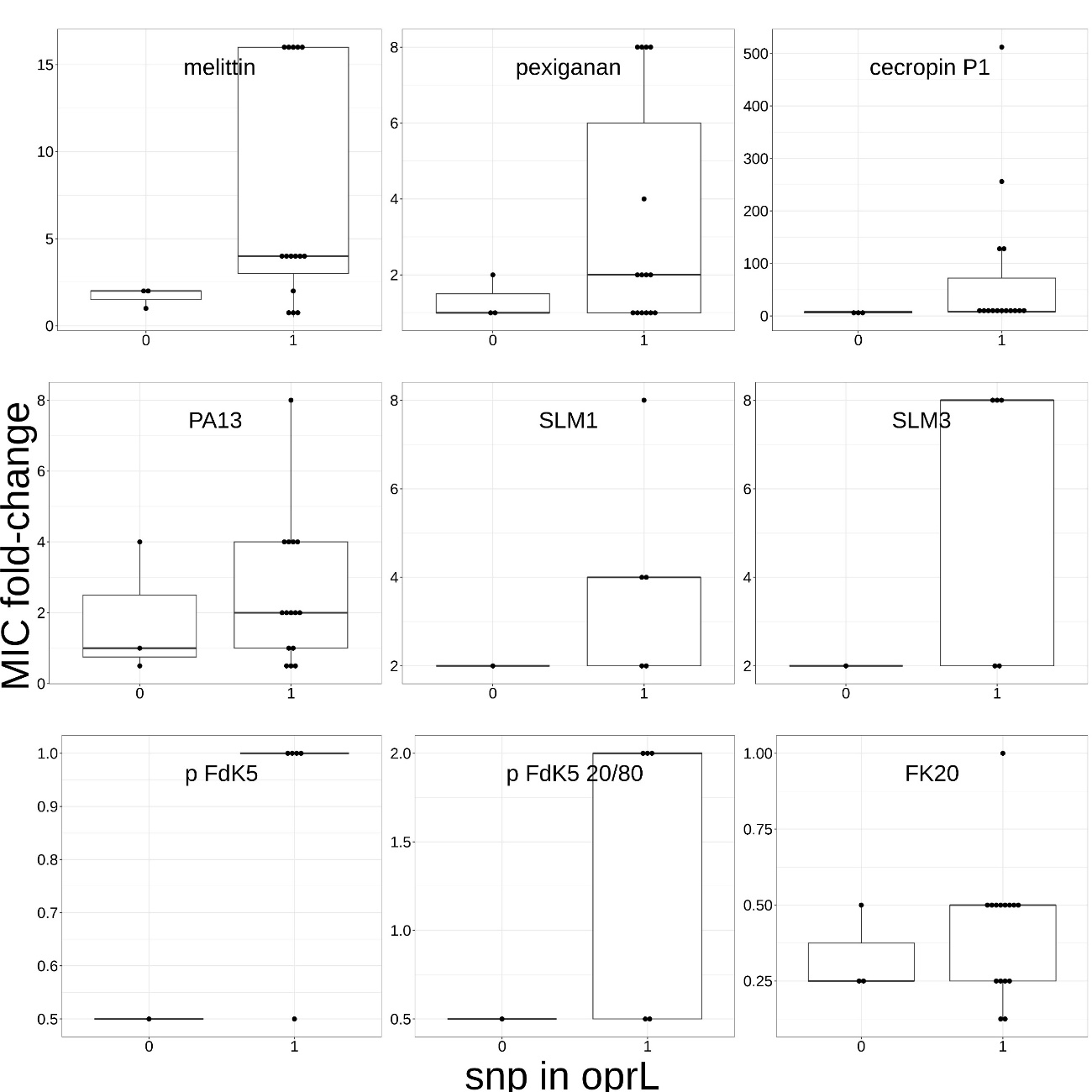


*Figure S9 – Boxplots representing the MIC fold-change towards several antimicrobials, according to the presence, or absence, of oprL gene SNPs (across strains from different selection regimes). The antimicrobial to which the bacterial strains were exposed to, is given in the window of the boxplot. On the x-axis: absence (0) or presence (1) of oprL gene SNPs. On the y axis: MIC fold-change of experimental evolution. The boxes span the range between the 25th and 75th percentile, while the horizontal black line inside represents the median value. The vertical bars extend to the minimum and maximum score, excluding outliers. The individual datapoints represent the MIC fold-change of 1 bacterial strain carrying a SNP. SNPs in the tpbB gene emerged only in 1 replicate of the experiment. The boxplots representing MIC fold changes towards Melittin, Pexiganan, Cecropin P1, PA13 and FK20 contain the data of strains originating from the Melittin, Pexiganan, Cecropin P1, PA13 and control selection regimes. The boxplots representing MIC fold-change towards SLM1, SLM3, p-FdK5 and p-FdK5 20/80 contain the data of strains originating from the SLM1 and control treatments. In the SLM1 selection regime, only 1 strain did not show a SNP in oprL, making the comparison between presence/absence of SNP irrelevant. The corresponding statistical tests are given in the Table S2. The data underlying this Figure can be found in* <https://doi.org/10.5281/zenodo.11209304>*.*
