# Supplementary material for: The evolution of antimicrobial peptide resistance in Pseudomonas aeruginosa is severely constrained by random peptide mixtures
Source: PLoS Biol. 2024 Jul 2;22(7):e3002692. doi: 10.1371/journal.pbio.3002692 (PMC11218975; doi:10.1371/journal.pbio.3002692)
Supplement: S13 Fig — The focal gene is given in the window of the boxplot. On the x-axis: absence (0) or presence (1) of SNPs in the gene. On the y-axis: lag time in minutes. The boxes span the range between the 25th and 75th percentile, while the horizontal black line inside represents the median value. The vertical bars extend to the minimum and maximum score, excluding outliers. The individual datapoints represent the lag time of 1 bacterial strain. The lag time shows less variation when SNPs are present in the lasR gene of the strains, and there is a strong trend for SNPs in the phoQ gene to increase the lag time of the strains (see main text for corresponding statistical tests). The data underlying this figure can be found in https://doi.org/10.5281/zenodo.11209304. (DOCX) [file pbio.3002692.s015.docx]

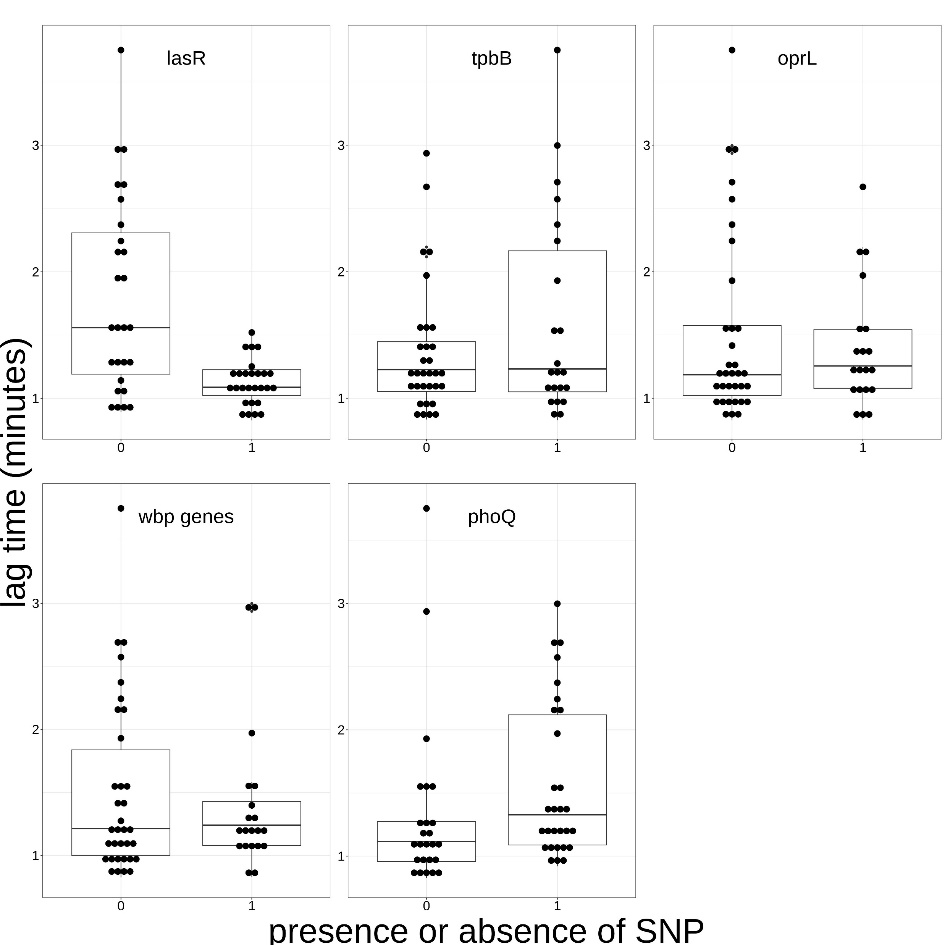


*Figure S13 – Boxplots representing the duration of the lag phase (lag time) in the presence or absence of SNP in the main 5 genes (5 genes harbouring most of the SNPs across strains from different selection regimes). The focal gene is given in the window of the boxplot. On the x-axis: absence (0) or presence (1) of SNPs in the gene. On the y-axis: lag time in minutes. The boxes span the range between the 25th and 75th percentile, while the horizontal black line inside represents the median value. The vertical bars extend to the minimum and maximum score, excluding outliers. The individual datapoints represent the lag time of 1 bacterial strain. The lag time shows less variation when SNPs are present in the lasR gene of the strains, and there is a strong trend for SNPs in the phoQ gene to increase the lag time of the strains (see main text for corresponding statistical tests). The data underlying this Figure can be found in* <https://doi.org/10.5281/zenodo.11209304>*.*
