# Supplementary material for: The evolution of antimicrobial peptide resistance in Pseudomonas aeruginosa is severely constrained by random peptide mixtures
Source: PLoS Biol. 2024 Jul 2;22(7):e3002692. doi: 10.1371/journal.pbio.3002692 (PMC11218975; doi:10.1371/journal.pbio.3002692)
Supplement: S1 Supplementary methods — (DOCX) [file pbio.3002692.s016.docx]

**Supplementary methods**

Modelling killing curves – Pharmacodynamics

To model the killing curve, the relationship between the concentration of AMPs/RPMs and the killing and/or growth rate of exposed bacteria, the Hill function was used (36):

$$\mu(a)=E_{\max}\frac{{(a/{EC}_{50})}^{\kappa}}{1+ {(a/{EC}_{50})}^{\kappa}} (1)$$

Here, µ(a) is the killing rate at a given concentration of AMPs/RPMs; a is a given concentration; E_max_ is the maximal killing rate of the given AMP/RPM. κ is the Hill coefficient. We then defined growth rate ψ(a) as follows:

$\psi\left( a \right)=\psi_{\max}-\mu(a)$ (2)

Here, ψ_max_ is the maximal growth rate of bacteria without AMPs/RPMs. The maximum effect of AMPs/RPMs is defined by:

$E_{\max}=\psi_{\max}-\psi_{\min}$ (3)

Thus, the effect of AMPs/RPMs in each concentration, µ(a), can be rewritten as:

$$\mu(a)=\frac{(\psi_{\max}-\psi_{\min}){(a/zMIC)}^{\kappa}}{{(a/zMIC)}^{\kappa}- \psi_{\min}/\psi_{\max}} (4)$$
